# Combined Radiofrequency Ablation and Left Atrial Appendage Closure in Atrial Fibrillation and Systolic Heart Failure

**DOI:** 10.3390/diagnostics13213325

**Published:** 2023-10-26

**Authors:** Jian Sun, Rui Zhang, Mei Yang, Wei Li, Peng-Pai Zhang, Bin-Feng Mo, Qun-Shan Wang, Mu Chen, Yi-Gang Li

**Affiliations:** Xinhua Hospital, School of Medicine, Shanghai Jiao Tong University, Shanghai 200092, China

**Keywords:** atrial fibrillation, catheter ablation, left atrial appendage closure, heart failure with reduced ejection fraction, heart failure with mildly reduced ejection fraction

## Abstract

Background: Managing patients with atrial fibrillation (AF) and comorbid heart failure (HF) with reduced (HFrEF) or mildly reduced ejection fraction (HFmrEF) is of clinical importance but a great challenge. This study aimed to evaluate the clinical benefit of the combined radiofrequency catheter ablation (RFCA) and left atrial appendage closure (LAAC) procedure in AF patients complicated with systolic HF. Methods: AF patients with HFrEF or HFmrEF who underwent the combined RFCA and LAAC procedure were prospectively enrolled in the LAACablation registry. The procedural complications and long-term outcomes were evaluated. Another cohort of AF patients with systolic HF who did not undergo either RFCA or LAAC were used for prognosis comparison. Results: Among 802 AF patients who underwent the combined procedure, 65 patients were comorbid with systolic HF (25 with HFrEF and 40 with HFmrEF). The overall procedural complication rate was 9.2%, which was mainly attributed to acute decompensated HF (6.2%). Accompanied with markedly reduced AF burden (from median [25th, 75th percentile]: 100 [100, 100] to 0 [0, 1.2]%, *p* < 0.001), upward trajectories of cardiac function were observed in 51 (78.4%) patients, showing improvement in New York Heart Classification (*p* < 0.01), natriuretic peptide levels (from 1492 [809, 3259] to 413 [163, 880] pg/mL, *p* < 0.001) and left ventricular EF (from 42.6 ± 5.3 to 53.8 ± 8.2%, *p* < 0.001). During the 27-month follow-up period, death, thromboembolism, major bleeding, and HF rehospitalization were observed in three, one, one, and four patients, respectively. The observed event rates showed a significant reduction compared with the non-procedure AF-HF cohort (*n* = 138; for composite endpoint: hazard ratio: 2.509, 95% confidence interval: 1.415–4.449, *p* = 0.002) and with the respective rates predicted by risk scores. Conclusions: Combining RFCA and LAAC achieves acceptable safety and credible long-term efficacy in AF patients with systolic HF. Further randomized studies are warranted in a larger patient cohort.

## 1. Introduction

Atrial fibrillation (AF) and heart failure (HF), increasingly common in the aging population, are frequently comorbid and beget each other [1,2,3]. When the two clinical conditions occur in one patient, risk of morbidity and mortality markedly increases [4]. On the one hand, AF worsens HF due to atrioventricular desynchrony, irregular ventricular rate causing inadequate left ventricular filling, ineffective atrial contraction, and arrhythmia-induced cardiomyopathy (AIC) [4]. Maintaining sinus rhythm by radiofrequency catheter ablation (RFCA) is beneficial to AF patients complicated with HF, which improves the survival rate, reduces re-hospitalization, and reverses cardiac dysfunction [5,6,7]. On the other hand, HF inversely begets AF, and aggregates the stroke risks in AF, which highlights the importance of stroke prophylaxis in this comorbid condition [8]. Despite a higher risk of thromboembolic complications, HF patients have much lower adherence to oral anticoagulation [9]. Therefore, the American Heart Association consensus suggests left atrial appendage (LAA) closure (LAAC) as an alternative treatment in patients with comorbid AF and HF with reduced ejection fraction (HFrEF) who have moderate to high stroke risks and contraindications to long-term oral anticoagulation [4]. However, the benefit of LAAC in this specific, high-risk population remains controversial [10]. Concerns are raised regarding unfavorably effects of LAAC on left atrial compliance and reservoir function, which might result in post-LAAC HF [11]. Other concerns include noticeable device-related thrombus (DRT), the ineffectiveness on left ventricular (LV) thrombus, and non-cardioembolic stroke facilitated by systemic hypercoagulable state in patients with LV systolic dysfunction [12,13].

The combined left atrial intervention of RFCA and LAAC has been demonstrated as a safe and effective therapeutic option to achieve rhythm control and stroke prevention simultaneously [14]. However, data regarding its application in AF patients complicated with HF are limited. The two processes of the combined procedure might compensate each other in the setting of AF-HF comorbidities. Theoretically, RFCA could improve the cardiac function, which consequently reduces the risks of post-LAAC HF, DRT and extra-LAA thrombi. LAAC provides stroke prophylaxis, as RFCA alone shows no clinically impactful reduction in thromboembolic risks [15]. In this proof-of-concept study, we reported the procedural safety and long-term effectiveness of the combined RFCA and LAAC in patients with AF-HF comorbidities who had reduced (HFrEF) or mildly reduced ejection fraction (HFmrEF).

## 2. Methods

### 2.1. Study Population

This study was approved by the ethics board of Xinhua Hospital and complies with the Declaration of Helsinki. Written informed consent was provided by all participants. A total of 802 AF patients who enrolled in the LAACablation registry were screened. The LAACablation registry (ClinicalTrials.gov ID: NCT03788941) is an observational single-center cohort study recruiting patients undergoing successful combined RFCA and LAAC procedures [16]. The detailed inclusion and exclusion criteria for the LAACablation registry were shown in Supplementary Table S1. The current study was the sub-analysis of the patients with systolic HF, which included (1) Patients presenting with HF symptoms and signs of fluid retention, such as fatigue, shortness of breath, dyspnea on exertion, orthopnea, paroxysmal nocturnal dyspnea, pulmonary congestion, and low extremity edema; (2) objective evidence of cardiac systolic dysfunction by transthoracic echocardiography (TTE). Systolic HF was set as HFrEF (≤40%) and HFmrEF (41–49%) of LV ejection fraction (LVEF) [17]. Patients without baseline TEE (*n* = 13), as well as with LVEF ≥ 50% (*n* = 724), were excluded. Therefore, we analyzed the remaining 65 patients with systolic HF who underwent successful procedures, including 25 HFrEF and 40 HFmrEF patients. 

In order to reveal the clinical benefit of the combined RFCA and LAAC procedure, we included another cohort of AF-HF patients who did not undergo the procedure for prognosis comparison. This cohort was derived from the patients who were admitted to the same institution during the same period. The inclusion criteria for this control cohort were as follows: (1) patients with HF symptoms and signs; (2) LVEF < 50% by TTE; (3) patients with documented AF; (4) patients who did not undergo RFCA or LAAC or the combined procedure. A total of 138 patients fulfilled the inclusion criteria and were followed. The inclusion time was set as the time of TTE revealing LV systolic dysfunction.

### 2.2. Guideline-Directed Medical Therapies

Guideline-directed medical therapies (GDMT) for HF, including beta-blockers, angiotensin-converting enzyme inhibitor (ACEi) or angiotensin II receptor blocker (ARB) or angiotensin receptor-neprilysin inhibitor (ARNi), mineralocorticoid receptor antagonist (MRA), and sodium-glucose co-transporter 2 inhibitor (SGLT2i), were continuously prescribed and optimized during the hospital stay [17]. For patients with New York Heart Association (NYHA) class III or ambulatory class IV, preprocedural HF management was intensified to guarantee procedural tolerance, including optimize volume status, intravenous diuretics and/or inotropic support as the “bridge therapy”.

### 2.3. A Combined Procedure of Catheter Ablation and LAAC

Within 48 h before the procedure, transesophageal echocardiography (TEE) was performed to exclude intracardiac thrombus, together with cardiac computed tomography angiography (CTA) if tolerated. HF patients underwent successive RFCA and LAAC, which were performed by experienced operators with an annual volume over 50 cases of the combined procedure. The procedures were uniformly conducted under conscious sedation. The CARTO navigation system (Biosense Webster, Diamond Bar, CA, USA) were used for atrial reconstruction and guidance of AF ablation. The THERMOCOOL SMARTTOUCH SF (STSF) catheter (Biosense Webster, Inc., Irvine, CA, USA) was used as its 56-hole tip irrigation facilitating cooling at low flow rate, thus easing the fluid management process. Pulmonary vein isolation (PVI) was performed in all patients. Additional ablation, including left atrial roof line, anterior septal, posterior and inferior lines, mitral isthmus (MI) and cavo-tricuspid isthmus (CTI) lines, complex fractionated electrograms (CFAE) modification, and ablation of ganglionated plexi and extra-PV triggers, were performed when deemed necessary. Vein of Marshall (VOM) ethanol infusion has also been conducted in selected patients. If sinus rhythm was not restored after ablation, low-energy intracardiac cardioversion up to 15 J, which was delivered between catheters positioned in the right atrium and the left atrium or the coronary sinus, was conducted. LAAC with WATCHMAN (Boston Scientific, Natick, MA, USA) device was subsequently performed under the guidance of fluoroscopy. Unless deemed necessary, the intraprocedural TEE was not performed to increase patients’ tolerance, shorten the procedural time, and avoid the anesthesia-related complications such as respiratory depression. The device deployed should fulfill the PASS (Position-Anchoring-Size-Seal) criteria evaluated by fluoroscopy [18]. Intravenous diuretics might be used intraprocedurally. 

### 2.4. Post-Procedure Management and Follow-Up

Patients were advised to outpatient follow-up every 3 months. TEE was scheduled at 3 months to detect any peri-device leaks (PDLs) and device-related thrombus (DRT). Since compliance for TEE was relatively poor in HF patients, an alternative cardiac CTA was performed to assess PDL and endothelialization of the device surface. Although indirect evaluation, endothelialization was defined by no contrast filling in LAA, while incomplete endothelialization as a complete or partial contrast filling. The TTE, 12-lead ECG, Holter monitoring, and GDMT optimization for HF were advised at every follow-up visit. As for the control cohort of patients who did not undergo the combined procedure, follow-up was conducted by clinic visit, telephone, and electronic medical records screening.

### 2.5. Events Definition

Procedural complications within 7 days were defined as acute decompensated HF, cardiac tamponade or pericardial effusion, device embolism, thromboembolism (stroke, transient ischemic attack [TIA] and systemic embolism), air embolism, major bleeding, and death. Adverse events during follow-ups included all-cause death, thromboembolic events (strokes, TIAs, and systemic embolisms), major bleeding and HF rehospitalization. In addition, recurrence of arrhythmias was evaluated and reported independently of other outcomes. Any atrial tachyarrhythmias, including AF, atrial flutter, or atrial tachycardia lasting more than 30 s after the 90-day blanking period, were considered a clinical recurrence.

### 2.6. Statistics

Continuous variables are presented as the mean ± standard deviation (SD), or median [25th, 75th percentile], and categorical variables as counts and percentages, unless specifically stated otherwise. Student’s *t* tests, Mann–Whitney tests, Pearson’s chi-square, and Fisher’s exact tests were used for comparisons between the HFrEF and HFmrEF groups as appropriate. Paired t tests were used for comparison of variables before and after procedure in same subjects. Cumulative event probabilities were estimated using the Kaplan–Meier method and the log-rank test to calculate the *p*-value. Observed rates of death, thromboembolic events, and major bleeding were compared to predicted mortality, thromboembolism, and bleeding rates derived from historical cohorts according to MAGGIC, CHA_2_DS_2_-VASc and HAS-BLED scores [19,20]. A two-sided *p*-value < 0.05 was considered statistically significant. Statistics were performed using SPSS 23.0 (IBM, Armonk, NY, USA).

## 3. Results

### 3.1. Patient Demographics

Among 802 patients who underwent a combined procedure of RFCA and LAAC in the LAACablation registry, 65 patients (8.1%) presented with with HF and LV systolic dysfunction, including 25 (3.1%) patients with HFrEF and 40 (5.0%) with HFmrEF. Patients’ baseline characteristics are summarized in Table 1. The patients had a mean age of 67.3 ± 7.5 years, with an average MAGGIC score of 20.1 ± 4.9, a CHA2DS2-VASc score of 3.9 ± 1.2, and a HAS-BLED score of 2.7 ± 1.0, respectively. Males (47, 67.3%), persistent or long-standing persistent AF (55, 84.6%), and NYHA class III (37, 56.9%), were dominant in terms of sexes, AF temporal types, and HF symptom classifications, respectively. Patients with AF-HF states were often comorbid with hypertension (51, 78.5%), diabetes (15, 23.1%) and prior stroke (13, 20.0%). A history of myocardial infarction was observed in 2 (3.1%) patients and major bleeding in 11 (16.9%) subjects. Compared with HFmrEF, HFrEF patients had lower LVEF (35.9 ± 4.2% vs. 45.6 ± 1.8%, *p* < 0.001) and a larger LV end-diastolic diameter (60.0 ± 8.5 mm vs. 55.7 ± 5.2 mm, *p* = 0.016), which accompanied with higher MAGGIC score (22.6 ± 5.1 vs. 19.0 ± 4.4, *p* = 0.006). Other characteristics were similar between the HFrEF and HFmrEF groups. Further, the baseline characteristics of the cohort who did not undergo the procedure (*n* = 138) were shown in Supplementary Table S2, showing similar demographic features with the patients who underwent the combined procedure.

### 3.2. Procedural and Index-Hospitalization Characteristics

As shown in Table 2, patient-tailored ablation strategies were adopted, with PVI performed and achieved in all patients. A various combination of linear ablation was added in 62 (95.4%) patients. Intracardiac cardioversion was performed in 20 (30.8%) subjects. Consequently, intraprocedural sinus rhythm restoration was achieved in all HF patients. Ablation strategies were similar between the HFrEF and HFmrEF groups, except for less mitral isthmus ablation in HFrEF (25.0% vs. 66.6%, *p* = 0.003). For the subsequent LAAC phase, cauliflower was the most common LAA morphology and 33 mm was the most selected device size for both groups. The total procedure time was 196 ± 50 min, including an ablation time of 62 ± 27 min and fluoroscopy time of 8.4 ± 4.5 min. The irrigated saline infusion volume was 1103 ± 423 mL.

The overall procedural complication rate was 9.2% and was comparable between the HFrEF and HFmrEF groups (10.0% vs. 8.8%, *p* > 0.999). The acute decompensated HF was the most prevalent complication (6.2%), which were all resolved by intravenous diuretics and/or inotropic support before discharge. In the HFmrEF group, there was one pericardial effusion not requiring pericardiocentesis and one transient air embolism during LAAC phase, respectively. No cardiac tamponade, device embolism, thromboembolic events, major bleeding, or death was observed in both groups. The index hospital stay was longer in the HFrEF than HFmrEF groups (11.8 ± 5.2 vs. 9.2 ± 3.3 days, *p* = 0.022), which was attributed to longer pre-procedural stay (6.6 ± 2.4 vs. 5.1 ± 2.3 days, *p* = 0.026).

### 3.3. Post-Procedural Management and Evaluation

The follow-up imaging evaluation at 3 months took places for all patients by TEE and/or cardiac CT (Table 3). Satisfactory seal (complete seal or PDL ≤ 5 mm) was achieved in all patients. The rate of complete endothelialization (no contrast filling in LAA) was achieved in 32.4% of patients evaluated by cardiac CT. DRT was found in three patients (one in HFrEF and two in HFmrEF).

The medication use was compared between baseline and 1-year after the procedure (Table 4). The number of patients taking anticoagulants significantly reduced from 86.1% pre-procedure to 12.3% at 1-year post-procedure, which was accompanied by increased number of patients on antiplatelet (from 10.8% to 70.8%), no antithrombotic therapy (from 3.1% to 16.9%). As for antiarrhythmic drugs, only an unsignificant downtrend of amiodarone was shown in HFrEF. The combination of GDMT for HF, including beta-blocker, ACEi/ARB/ARNi, MRA and SGLT2i, was comparable between pre- and post- procedure. However, up-titration of beta-blocker and renin–angiotensin–aldosterone system inhibitors were noticed in HFrEF patients (from 20% to 60%, *p* = 0.010, and from 5% to 55%, *p* = 0.001, respectively). The use of diuretics remained similar; however, digitalis was less prescribed after procedure (from 50% to 20%, *p* = 0.041 for HFrEF, and from 28.9% to 8.9%, *p* = 0.029 for HFmrEF).

**Figure 1:**
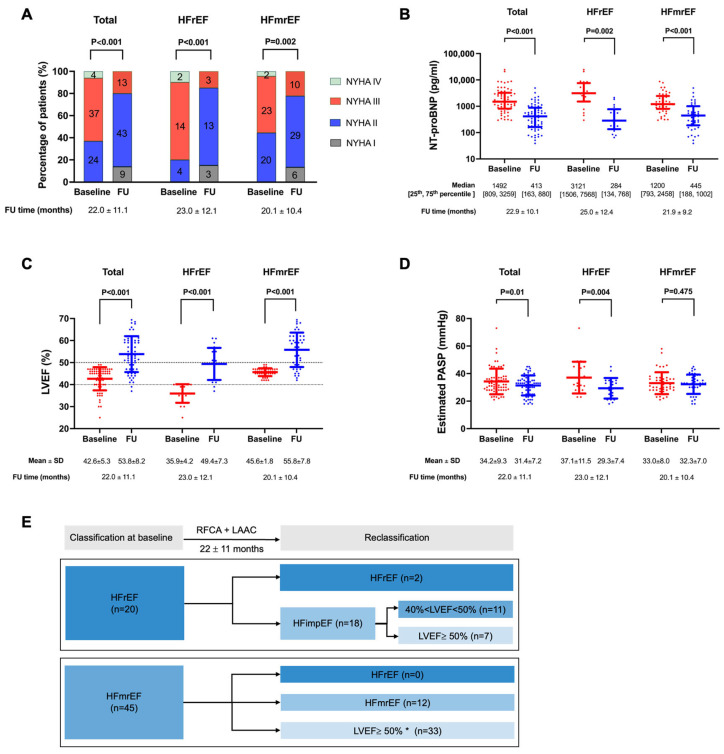
Heart failure reevaluation during follow-up in patients who underwent the combined procedure. (**A**). NYHA classification. (**B**). NT-proBNP. Y axis is scaled to log 10. (**C**). LVEF. (**D**). Estimated PASP. (**E**). Trajectory of heart failure. Bars show median and 25th and 75th percentile in (**B**), and mean and SD in (**C**,**D**). * Limited evidence to guide whether to treat these patients as HFpEF or HFmrEF. FU = follow-up; HFimpEF = heart failure with improved ejection fraction; HFmrEF = heart failure with mildly reduced ejection fraction; HFrEF = heart failure with reduced ejection fraction; LAAC = left atrial appendage closure; LVEF = left ventricular ejection fraction; NT-proBNP = N-terminal-proB-type natriuretic peptide; NYHA = New York Heart Association; PASP = pulmonary arterial systolic pressure; RFCA = radiofrequency catheter ablation.

Serial assessments of HF were performed during the follow-up, and the data of last time reevaluation was used for analyses (Figure 1). The NYHA classification was significantly improved for both groups. The value of N-terminal-proB-type natriuretic peptide (NT-proBNP) was reduced (from 3121 [1506, 7568] to 284 [134, 768], *p* = 0.002 for HFrEF; from 1200 [793, 2458] to 445 [188, 1002], *p* < 0.001 for HFmrEF). The follow-up TTE revealed markedly improvement of LVEF in both HFrEF (35.9 ± 4.2% vs. 49.4 ± 7.3%, *p* < 0.001) and HFmrEF (45.6 ± 1.8% vs. 55.8 ± 7.8%, *p* < 0.001), along with a reduction in estimated pulmonary arterial systolic pressure (PASP) in HFrEF (37.1 ± 11.5 vs. 29.3 ± 7.4 mmHg, *p* = 0.004). Consequently, upward trajectories of HF reclassification based on LVEF were observed in 18 (90.0%) patients with HFrEF and 33 (73.3%) patients with HFmrEF at 22 ± 11 months.

### 3.4. Events Follow-Up

With an average of 27.4 ± 7.5 months follow-up, death was observed in three patients (one HFrEF and two HFmrEF), including two cardiovascular deaths and one death from pancreatic cancer. One stroke and one major bleeding were observed in the HFmrEF group, respectively. HF rehospitalization was observed in four patients (two for each group). The rates of each event and the composite were similar between the HFrEF and HFmrEF groups (Supplementary Figure S1). 

**Figure 2:**
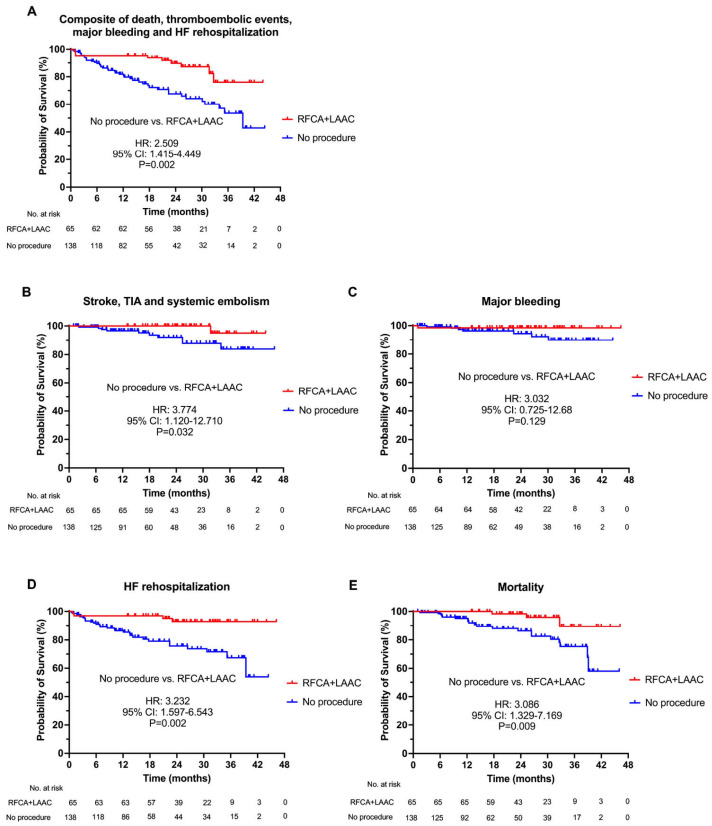
Time-to-event curves comparing AF patients with systolic HF underwent the combined RFCA + LAAC procedure and no procedure. (**A**). Cumulative incidence of the composite of thromboembolic events, major bleeding, heart failure rehospitalization, and death. (**B**). Cumulative incidence of thromboembolic events (strokes, TIA, and systemic embolism). (**C**). Cumulative incidence of major bleeding. (**D**). Cumulative incidence of heart failure rehospitalization. (**E**). Cumulative incidence of mortality. Red curves were derived from the LAACablation registry and blue curves were derived from the control cohort of patients who did not underwent RFCA, or LAAC, or the combined procedure. Patients with HFrEF and HFmrEF were pooled. AF = atrial fibrillation; CI = confidence interval; HF = heart failure; HFmrEF = heart failure with mildly reduced ejection fraction; HFrEF = heart failure with reduced ejection fraction; HR = hazard ratio; LAAC = left atrial appendage closure; RFCA = radiofrequency catheter ablation; TIA = transient ischemic attack.

**Figure 3:**
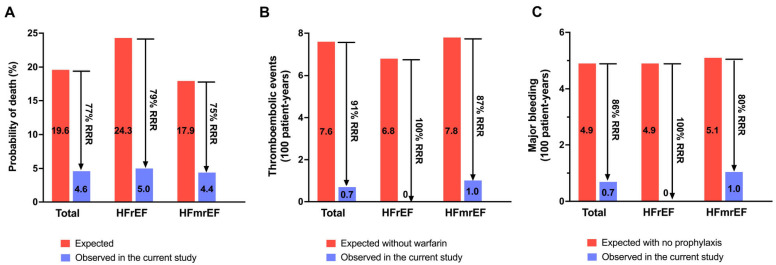
Relative risk reduction by the combined procedure comparing with the expected rates. (**A**). Rates of death in the cohort compared to expected death probabilities according to MAGGIC score for predicting survival in heart failure at 27.4 months (mean follow-up time). (**B**). Thromboembolic events (strokes, transient ischemic attacks, and systemic embolism) rate per 100 patient-years in the cohort of the combined procedure compared to expected rates when taking warfarin according to CHA_2_DS_2_-VASc score. (**C**). Major bleeding rates per 100 patient-years in the cohort of the combined procedure compared to expected rates according to HAS-BLED score. CHA_2_DS_2_-VASc = Congestive Heart Failure, Hypertension, Age ≥ 75 [Doubled], Diabetes Mellitus, prior stroke or Transient Ischemic Attack [Doubled], Vascular Disease, Age 65–74, Female; CRT-D = cardiac resynchronization therapy with defibrillation; HAS-BLED = hypertension, abnormal renal and/or liver function, previous stroke, bleeding history or predisposition, labile international normalized ratios, elderly, and concomitant drugs and/or alcohol excess; HFmrEF = heart failure with mildly reduced ejection fraction; HFrEF = heart failure with reduced ejection fraction; MAGGIC = Meta-Analysis Global Group in Chronic Heart Failure; RRR = relative risk reduction.

In comparison, the numbers of death, thromboembolism, major bleeding, and HF rehospitalization events in the non-procedural AF-HF cohort were 20, 10, 7, and 29, respectively. Compared with non-procedural cohort of AF-HF (including both HFrEF and HFmrEF) patients (Figure 2), patients who underwent the combined RFCA and LAAC procedure showed significantly lower incidences of composite adverse events (RFCA + LAAC vs. no procedure: HR = 2.509, 95% CI = 1.415–4.449, *p* = 0.002), as well as the components including thromboembolic events (HR = 3.774, 95% CI = 1.120–12.710, *p* = 0.032), HF rehospitalization (HR = 3.232, 95% CI = 1.597–6.543, *p* = 0.002), and mortality (HR = 3.086, 95% CI = 1.329–7.169, *p* = 0.009), respectively. The rates of major bleeding were comparable between patients with or without the procedure. In addition, the comparisons between the procedural and non-procedural cohorts in AF-HFrEF and AF-HFmrEF patients were shown in Supplementary Figures S2 and S3, respectively. When comparing with the risk score prediction, the combined procedure was associated with 77%, 91% and 86% risk reduction in mortality, thromboembolic events and major bleeding relative to the predicted rates by MAGGIC, CHA_2_DS_2_-VASc and HAS-BLED scores, respectively (Figure 3) [19,20]. 

**Figure 4:**
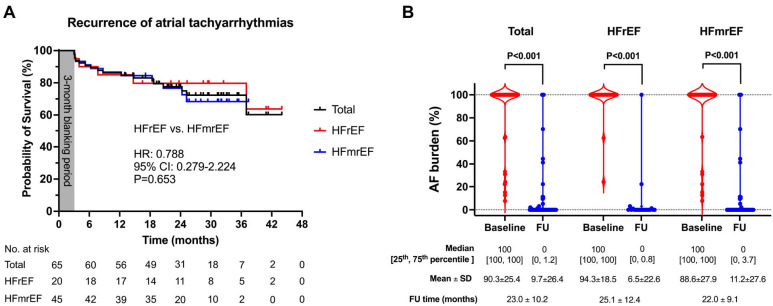
Recurrence and burden of atrial tachyarrhythmias after the combined RFCA + LAAC procedure. (**A**). Recurrence of atrial tachyarrhythmia over time after the combined procedure. Shown are Kaplan–Meier estimates of the recurrence of any atrial tachyarrhythmia (AF, atrial flutter, or atrial tachycardia) lasting at least 30 s. A 90-day blanking period was adopted. Tick marks indicate censored data. The comparison was made between patients with HFrEF and HFmrEF. (**B**). AF burden (including AF, atrial flutter, or atrial tachycardia) at 24-h Holter monitoring at baseline and during the follow-up. AF, atrial fibrillation; CI = confidence interval; FU = follow-up; HFmrEF = heart failure with mildly reduced ejection fraction; HFrEF = heart failure with reduced ejection fraction; HR = hazard ratio; SD = standard deviation.

The recurrent atrial tachyarrhythmias after the procedure were observed in 17 patients (5 in HFrEF and 12 in HFmrEF). The rates of recurrence were comparable between groups (hazard ratio: 0.788, 95% confidence interval: 0.279–2.224, *p* = 0.653, Figure 4). The burden of atrial tachyarrhythmias significantly reduced in both the HFrEF (from 100 [100, 100] to 0 [0, 0.8], *p* < 0.001) and HFmrEF (from 100 [100, 100] to 0 [0, 3.7], *p* < 0.001) groups. 

## 4. Discussion

### 4.1. Main Findings

The proof-of-concept study investigated the procedural safety and long-term effectiveness of the combined RFCA and LAAC in AF patients, complicated with HFrEF or HFmrEF. The main findings were as follows. (1) The procedural safety was acceptable, and acute decompensated HF was the main procedure-related complication. (2) The combined procedure, along with optimized GDMT, improves cardiac systolic function in the majority of patients. (3) The combined procedure might reduce the risks of mortality and thromboembolism and ameliorates AF burden. Therefore, the combined RFCA and LAAC is a feasible approach in treating patients with AF and systolic HF. 

### 4.2. Effects of Catheter Ablation and Left Atrial Appendage Closure on Cardiac Function

The impacts of LAAC on cardiac function remained controversial and were not consistently assessed in the previous trials of LAAC [21,22,23]. Exclusion of LAA might impair the reservoir, booster-pump, and hormonal functions of LA, resulting in increased vulnerability to volume overload and unfavorable hemodynamics in both LV and pulmonary circulation. LAAC also led to functional and structural remodeling of LA and LV, which might further facilitate the maintenance of AF and deterioration of HF [24]. Despite neutral effects on HF biomarkers, LAAC might lead to more frequent HF hospitalization, especially in patients with HF history [25].

The potentially negative effects of LAAC on cardiac function could be compensated or even reversed when combined with RFCA. The impact of LAAC-induced reservoir impairment might be minimized by the improved LA contractile, promoted atrioventricular synchrony, eliminated rhythm irregularity, and elevated LVEF if sinus rhythm was restored by RFCA. Over time, functional and structural reverse remodeling of LA and LV took places, which were especially prominent in those with systolic HF [11,26]. This was consistent with the results from the current study in both HFrEF and HFmrEF patients, that improvement of NYHA classification, NT-proBNP level, and LVEF were observed along with markedly reduced AF burden. Consequently, the risks of HF rehospitalization and mortality were ameliorated. The optimization of GDMT might also contribute, as there were more up-titration of beta-blocker and renin-angiotensin–aldosterone system inhibitors after the procedure. GDMT per se not only improved HF outcomes, but also served as an upstream therapy to reduce AF risks [27]. Nevertheless, restoration of sinus rhythm by RFCA might facilitate the achievement of maximal tolerance to GDMT, as the up-titration could be restricted by unstable hemodynamics during AF.

Nevertheless, procedure-related acute HF was still noticeable, the rate of which was comparable with previous studies reporting AF RFCA in HF [28]. Acute HF might be correlated with intraprocedural saline infusion, post-ablation atrial stunning and LAAC-induced sudden hemodynamic changes. Serial measures have been taken to lower the incidence of periprocedural HF. The preprocedural HF management included careful evaluation of the patients’ tolerance to procedure, volume status optimization, continuous use of GDMT, intravenous diuretics and/or inotropic support if deemed necessary. Such management led to longer preprocedural stay in the HFrEF than HFmrEF groups. The procedure was performed under conscious sedation and without TEE guidance to increase patients’ tolerance and reduce the anesthetic complications. The 56-pole ablation catheter was solely used to reduce the irrigated saline infusion. Less lateral mitral isthmus ablation was performed in HFrEF to shorten the procedure time, since the anterior septal wall was usually accompanied with more low voltage areas and anterior septal line could also block the peri-mitral reentrant. In addition, post-procedural fluid was also strictly controlled and intravenous diuretics might be used when necessary. 

### 4.3. The Combined Procedure on Stroke Prevention

For AF patients complicated with HF, up-to-date randomized data suggested RFCA rhythm control was associated with significantly lower all-cause mortality, reduced re-hospitalization rate, greater improvement in LVEF, but similar rate of stroke events, which might be attributed to asymptomatic AF recurrence, atrial cardiomyopathy and non-cardioembolic strokes [7,15]. Therefore, anticoagulation according to CHA_2_DS_2_-VASc scores was still required after “successful” RFCA. The stroke risks were intensified when complicated with HF, as well as with comorbidities predisposing both AF and HF, such as hypertension and diabetes [8]. However, HF patients tended to have lower adherence to oral anticoagulation and might be more vulnerable to bleeding events when AF was sustained [9,29]. Furthermore, LAA thrombus was prevalent ranging between 40% and 68% in HF patients with sinus rhythm [12]. Taken together, LAAC was a reasonable choice for stroke prevention in AF-HF comorbidities, even when sinus rhythm was restored by RFCA [4].

However, controversies exist regarding the efficacy of LAAC in this specific population. HF per se served as a substrate for a thromboembolic state due to stasis of flow, hypercoagulability, and endothelial dysfunction [12]. Such thromboembolic events were not only attributed to LAA-derived thromboembolism, but also included LV thrombi, LA chamber thrombi, deep venous thrombosis, pulmonary embolism, and myocardial infarction [12]. The latter events were not preventable by the local therapy of LAA. However, RFCA of AF reversed AIC, improved LVEF, led to reverse remodeling of both LA and LV, and consequently ameliorated the static hemodynamic states during HF. This concept was supported by the current study observing a low rate of residual stroke accompanied by sinus rhythm and LV function restoration. Nevertheless, DRT was noticeable, requiring prolonged anticoagulation, which was consistent with previous reports of higher DRT incidence associated with HF and non-paroxysmal AF [13]. 

## 5. Limitations

This study was an observational study, lacking a randomized control group (such as a cohort of patients who underwent RFCA only), which limited the ability to establish strong causal relationships. Therefore, this study may only serve as a proof-of-concept study, rather than a claim of direct clinical implications. Although it is derived from a large cohort of the combined RFCA and LAAC procedure, the number of HFrEF and HFmrEF patients in this study was relatively small. The results required verification by a larger sample size with longer follow-up period. The patients in the current study mainly presented with persistent AF and non-ischemic etiology, and were more likely to have ameliorated AF burden, reversable LV function, and less extra-LAA thrombi after procedure. Whether the combined procedure benefits HF patients with lower baseline AF burden and ischemic heart diseases remains unclear. Patients with extremely low LVEF (<25%) were not enrolled in this study, as they usually presented with worse prognosis [30]. As a comprehensive therapeutic approach, all procedures in this study were performed by experienced operators to guarantee procedural safety, time-efficiency, and effectiveness. A longer learning curve might be required when performing procedures in patients with severe HF.

## 6. Conclusions

The proof-of-concept study found that the combined RFCA and LAAC procedure achieves acceptable safety and credible long-term efficacy in AF patients complicated with systolic HF. Further randomized studies are warranted in a larger patient cohort.

## Figures and Tables

**Table 1:** Baseline characteristics of patients who underwent the combined procedure.

Patient Characteristics	Total (*n* = 65)	HFrEF (*n* = 20)	HFmrEF (*n* = 45)	*p*-Value
Age	67.3 ± 7.5	67.3 ± 9.8	67.3 ± 6.4	0.967
Age < 65 y	17 (26.2)	4 (20.0)	13 (28.9)	0.551
65 y ≤ age < 75 y	36 (55.4)	11 (55.0)	25 (55.6)	0.967
Age ≥ 75 y	12 (18.5)	5 (25.0)	7 (15.5)	0.490
Female	18 (27.7)	6 (30.0)	12 (26.6)	0.782
BMI (kg/m^2^)	25.7 ± 3.4	25.1 ± 4.2	26.0 ± 3.1	0.339
AF types				
Paroxysmal	10 (15.4)	3 (15.0)	7 (15.6)	>0.999
Persistent/long-standing persistent	55 (84.6)	17 (85.0)	38 (84.4)	>0.999
CHA_2_DS_2_-VASc score	3.9 ± 1.2	3.6 ± 1.3	4.0 ± 1.1	0.225
HAS-BLED score	2.7 ± 1.0	2.7 ± 1.3	2.8 ± 1.0	0.848
MAGGIC score *	20.1 ± 4.9	22.6 ± 5.1	19.0 ± 4.4	**0.006**
NYHA class				
NYHA class II	24 (36.9)	4 (20.0)	20 (44.4)	0.094
NYHA class III	37 (56.9)	14 (70.0)	23 (51.1)	0.156
Ambulatory NYHA class IV	4 (6.2)	2 (10.0)	2 (4.4)	>0.999
Hypertension	51 (78.5)	16 (80.0)	35 (77.8)	>0.999
Diabetes mellites	15 (23.1)	3 (15.0)	12 (26.7)	0.359
Myocardial infarction	2 (3.1)	0 (0)	2 (4.4)	>0.999
Cardiac revascularization ^†^	6 (9.2)	1 (5.0)	5 (11.1)	0.657
Hypertrophic cardiomyopathy	4 (6.2)	2 (10.0)	2 (4.4)	0.581
Left ventricular non-compaction cardiomyopathy	2 (3.1)	0 (0)	2 (4.4)	>0.999
Obstructive sleep apnea	4 (6.2)	3 (15.0)	1 (2.2)	0.083
Chronic obstructive pulmonary disease	3 (4.6)	2 (10.0)	1 (2.2)	0.222
History of strokes/TIAs/SE	13 (20.0)	2 (10.0)	11 (24.4)	0.314
History of LAA thrombus	6 (9.2)	2 (10.0)	4 (8.9)	>0.999
History of major bleeding	11 (16.9)	4 (20.0)	7 (15.6)	0.726
History of malignant tumor	4 (6.2)	1 (5.0)	3 (6.7)	>0.999
History of chemotherapy	3 (4.6)	1 (5.0)	2 (4.4)	>0.999
Pacemaker implantation	3 (4.6)	1 (5.0)	2 (4.4)	>0.999
ICD/RT-D implantation	2 (3.1)	1 (5.0)	1 (2.2)	0.524
Current smoker	23 (35.4)	9 (45.0)	14 (31.1)	0.400
Echocardiographic parameters				
LVEF (%)	42.6 ± 5.3	35.9 ± 4.2	45.6 ± 1.8	**<0.001**
Left atrial diameter (mm)	47.1 ± 5.0	46.5 ± 5.5	47.4 ± 4.9	0.532
LVEDD (mm)	57.0 ± 6.6	60.0 ± 8.5	55.7 ± 5.2	**0.016**
LVESD (mm)	44.1 ± 6.6	48.9 ± 8.1	42.0 ± 4.5	<0.001
Estimated PASP (mmHg)	34.3 ± 9.3	37.1 ± 11.5	33.0 ± 8.0	0.106
Pericardial effusion, mm ^‡^	2.6 ± 1.2	2.9 ± 1.1	2.5 ± 1.3	0.222
NT-proBNP (pg/mL)	1492 [809, 3259]	3121 [1506, 7568]	1200 [793, 2458]	**0.006**
Troponin I (ng/mL)	0.019 [0.009, 0.033]	0.028 [0.009, 0.054]	0.018 [0.009, 0.029]	0.313
Hemoglobin, g/L	136 ± 14	138 ± 16	135 ± 14	0.398
Creatine, mmol/L	76 ± 19	76 ± 16	76 ± 20	0.903

Values are given as the mean ± SD, median [25th percentile, 75th percentile], or *n* (%) as appropriate. * MAGGIC score for predicting survival in heart failure [19] was calculated by http://www.heartfailurerisk.org, (the date of patient enrollment). ^†^ including percutaneous transluminal coronary angioplasty and/or coronary artery bypass grafting. ^‡^ evaluating the depth of the echo-free space. AF = atrial fibrillation; BMI = body mass index; CHA_2_DS_2_-VASc = Congestive Heart Failure, Hypertension, Age ≥ 75 [Doubled], Diabetes Mellitus, prior stroke or transient ischemic attack [Doubled], Vascular Disease, Age 65–74, Female; CRT-D = cardiac resynchronization therapy with defibrillation; HAS-BLED = hypertension, abnormal renal and/or liver function, previous stroke, bleeding history or predisposition, labile international normalized ratios, elderly, and concomitant drugs and/or alcohol excess; ICD = implantable cardioverter defibrillator; LAA = left atrial appendage; LVEDD = left ventricular end-diastolic diameter; LVEF = left ventricular ejection fraction; LVESD = left ventricular end-systolic diameter; MAGGIC = Meta-Analysis Global Group in Chronic Heart Failure; NT-proBNP = N-terminal-proB-type natriuretic peptide; NYHA = New York Heart Association; PASP = pulmonary artery systolic pressure; SE = systemic embolism; TIA = transient ischemic attack.

**Table 2:** Procedural and index-hospitalization characteristics of patients who underwent the combined procedure.

Characteristics	Total (*n* = 65)	HFrEF (*n* = 20)	HfmrEF (*n* = 45)	*p*-Value
Ablation phase				
PVI (total)	65 (100)	20 (100.0)	45 (100)	>0.999
PVI only	3 (4.6)	1 (5.0)	2 (4.4)	>0.999
LA roof line	50 (76.9)	15 (75.0)	35 (77.7)	>0.999
LA posterior and/or inferior lines	33 (50.8)	9 (45.0)	24 (53.3)	0.535
Posterior wall isolation	27 (41.5)	7 (35.0)	20 (44.4)	0.476
Anterior septal line	41 (63.1)	12 (60.0)	29 (64.4)	0.785
Mitral isthmus line	35 (53.8)	5 (25.0)	30 (66.6)	**0.003**
CS and GCV musculature ablation	10 (15.4)	1 (5.0)	9 (20.0)	0.156
VOM ethanol infusion	10 (15.4)	2 (10.0)	8 (17.7)	0.711
LAA isolation	5 (7.7)	1 (5.0)	4 (8.9)	>0.999
Cavo-tricuspid line	48 (73.8)	12 (60.0)	36 (80.0)	0.127
Superior vena cava isolation	3 (4.6)	1 (10.0)	2 (4.4)	>0.999
CFAE ablation	39 (60.0)	11 (55.0)	27 (60.0)	0.706
Ganglionated plexi ablation	1 (1.5)	0 (0)	1 (2.2)	>0.999
Intracardiac cardioversion	20 (30.8)	7 (35.0)	13 (28.8)	0.622
Intraprocedural sinus rhythm restoration	65 (100.0)	20 (100.0)	45 (100)	>0.999
LAAC phase				
LAA morphology types				
Chicken wing	17 (26.2)	8 (40.0)	9 (20.0)	0.090
Windsock	5 (7.7)	1 (5.0)	4 (8.9)	>0.999
Cauliflower	40 (61.5)	10 (50.0)	30 (66.7)	0.202
Cactus	3 (4.6)	1 (5.0)	2 (4.4)	>0.999
LAA ostium, mm	23.8 ± 3.5	24.1 ± 3.9	23.7 ± 3.3	0.648
Device size for WATCHMAN				
21 mm	4 (6.2)	2 (10.0)	2 (4.4)	0.581
24 mm	11 (16.9)	2 (10.0)	9 (20.0)	0.480
27 mm	16 (24.6)	6 (30.0)	10 (22.2)	0.502
30 mm	12 (18.5)	2 (10.0)	10 (22.2)	0.315
33 mm	22 (33.8)	8 (40.0)	14 (31.1)	0.485
Total procedure time, min	196 ± 50	183 ± 55	203 ± 46	0.133
Ablation time, min	62 ± 27	56 ± 26	64 ± 27	0.269
Fluoroscopy time, min	8.4 ± 4.5	8.1 ± 4.7	8.6 ± 4.1	0.666
Irrigation fluid volume, ml	1103 ± 423	1035 ± 432	1147 ± 417	0.303
Procedural complications (total)	6 (9.2)	2 (10.0)	4 (8.8)	>0.999
Acute decompensated heart failure	4 (6.2)	2 (10.0)	2 (4.4)	0.581
Cardiac tamponade	0 (0)	0 (0)	0 (0)	>0.999
Pericardial effusion not requiring pericardiocentesis	1 (1.5)	0 (0)	1 (2.2)	>0.999
Device embolism	0 (0)	0 (0)	0 (0)	>0.999
Stroke/TIA/systemic embolism	0 (0)	0 (0)	0 (0)	>0.999
Air embolism	1 (1.5)	0 (0)	1 (2.2)	>0.999
Major bleeding	0 (0)	0 (0)	0 (0)	>0.999
Death	0 (0)	0 (0)	0 (0)	>0.999
Total hospital stay, days	10.0 ± 4.1	11.8 ± 5.2	9.2 ± 3.3	**0.022**
Pre-procedural hospital stay, days	5.6 ± 2.4	6.6 ± 2.4	5.1 ± 2.3	**0.026**
Post-procedural hospital stay, days	4.4 ± 3.0	5.2 ± 4.7	5.2 ± 4.7	0.173

AF = atrial fibrillation; CFAE = complex fractionated atrial electrogram; CTI = cavo-tricuspid isthmus; CS = coronary sinus; GCV = great cardiac vein; HFmrEF = heart failure with mildly reduced ejection fraction; HFrEF = heart failure with reduced ejection fraction; LA = left atrial; LAA = left atrial appendage; LAAC = left atrial appendage closure; PVI = pulmonary vein isolation; PW = posterior wall; SVC = superior vena cava; TIA = transient ischemic attack; VOM = vein of Marshall.

**Table 3:** Post-procedural imaging evaluation of patients who underwent the combined procedure.

Post-Procedural Imaging	Total (*n* = 65)	HFrEF (*n* = 20)	HFmrEF (*n* = 45)	*p*-Value
TEE evaluation, *n*	46 (70.8)	15 (75.0)	31 (68.9)	0.770
Compression ratio, %	15.4 ± 2.3	16.0 ± 2.4	15.2 ± 2.2	0.216
DRT	3 (6.5)	1 (6.7)	2 (6.5)	>0.999
No PDL ^†^	33 (71.7)	12 (80.0)	21 (67.7)	0.497
PDL ≤ 5 mm	13 (28.3)	3 (0.2)	10 (32.3)	0.497
PDL > 5 mm	0 (0)	0 (0)	0 (0)	>0.999
Satisfactory seal	46 (100)	15 (100)	31 (100)	>0.999
Cardiac CT evaluation, *n*	37 (56.9)	12 (60.0)	25 (55.6)	0.738
LAA—no contrast filling ^‡^	12 (32.4)	4 (33.3)	8 (32.0)	>0.999
LAA—contrast filling	25 (67.6)	8 (66.7)	17 (68.0)	>0.999
Contrast filling with visible PDL	19(51.4)	5 (41.7)	13 (52.0)	>0.999
Contrast filling without PDL	6 (16.2)	2(17.7)	4(16.0)	>0.999

All patients who underwent 3-month post-procedural imaging evaluation. Among them, 18 patients underwent both TEE and CT evaluations. ^†^ Also known as complete seal. ^‡^ Suggesting a complete endothelialization. CT, computed tomography; DRT, device-related thrombus; HFmrEF = heart failure with mildly reduced ejection fraction; HFrEF = heart failure with reduced ejection fraction; LAA, left atrial appendage; PDL, peri-device leak; TEE, transesophageal echocardiography.

**Table 4:** Medication before and at 1-year post-index procedure of patients who underwent the combined procedure.

Oral Medications	HFrEF (*n* = 20)			HFmrEF (*n* = 45)		
	Before	1-Year	*p*-Value	Before	1-Year	*p*-Value
Antithrombotic regimen						
Oral anticoagulants	17 (85.0)	3 (15.0)	**<0.001**	39 (86.7)	5 (11.1)	**<0.001**
Warfarin	6 (30.0)	0 (0)	**0.020**	11 (24.4)	1 (2.2)	**0.004**
NOAC	11 (55.0)	3 (15.0)	**0.019**	28 (62.2)	4 (8.9)	**<0.001**
Antiplatelets	2 (10.0)	14 (70.0)	**<0.001**	5 (11.1)	32 (71.1)	**<0.001**
Dual antiplatelets	1 (5.0)	0 (0)	>0.999	2 (4.4)	1 (2.2)	>0.999
Single antiplatelet	1 (5.0)	14 (70.0)	**<0.001**	3 (6.7)	31 (68.9)	**<0.001**
None	1 (5.0)	3 (15.0)	0.605	1 (2.2)	8 (17.8)	**0.030**
Antiarrhythmic drugs	8 (40.0)	3 (15.0)	>0.999	6 (13.3)	3 (6.7)	0.485
Class I *	0 (0)	0 (0)	>0.999	0 (0)	0 (0)	>0.999
Amiodarone	8 (40.0)	2 (10.0)	0.065	5 (11.1)	3 (6.7)	0.714
Other Class III ^†^	0 (0)	0 (0)	>0.999	1 (2.2)	0 (0)	>0.999
Class IV	0 (0)	0 (0)	>0.999	0 (0)	0 (0)	>0.999
Medications for heart failure						
Beta-blocker	19 (95.0)	19 (95.0)	>0.999	28 (62.2)	33 (73.3)	0.259
Initiation dosage	15 (75.0)	7 (35.0)	**0.025**	22 (48.9)	24 (53.3)	0.673
Up-titration	4 (20.0)	12 (60.0)	**0.010**	6 (13.3)	9 (20.0)	0.396
ACEi/ARB/ARNi ^‡^	16 (80.0)	18 (90.0)	0.661	27 (60.0)	26 (57.8)	>0.999
Initiation dosage	15 (75.0)	7 (35.0)	**0.010**	24 (53.3)	21 (46.7)	0.527
Up-titration	1 (5.0)	11 (55.0)	**0.001**	3 (6.7)	5 (11.1)	0.288
MRA	18 (90.0)	17 (85.0)	>0.999	24 (53.3)	23 (51.1)	>0.999
Initiation dosage	16 (80.0)	15 (75.0)	>0.999	23 (51.1)	21 (46.7)	0.673
Up-titration	2 (10.0)	2 (10.0)	>0.999	1 (2.2)	2 (4.4)	>0.999
SGLT2i ^§^	1 (5.0)	3 (15.0)	0.605	2 (4.4)	4 (8.9)	0.677
Combination of GDMT ^¶^						
No therapy	0 (0)	0 (0)	>0.999	4 (8.9)	3 (6.7)	>0.999
Single therapy	0 (0)	0 (0)	>0.999	15 (33.3)	11 (24.4)	0.352
Double therapy	7 (35.0)	4 (20.0)	0.480	12 (26.7)	18 (40.0)	0.180
Triple therapy	12 (60.0)	15 (75.0)	0.501	14 (31.1)	13 (28.9)	0.929
Quadruple therapy	1 (5.0)	1 (5.0)	>0.999	0 (0)	0 (0)	>0.999
Diuretics	18 (90.0)	17 (85.0)	>0.999	32 (71.1)	28 (62.2)	0.371
Digitalis	10 (50.0)	3 (20.0)	**0.041**	13 (28.9)	4 (8.9)	**0.029**
Ivabradine	0 (0)	0 (0)	>0.999	0 (0)	1 (2.2)	>0.999

* including propafenone, mexiletine, and moricizine. ^†^ including sotalol and dronedarone. ^‡^ ARNi was covered by medical insurance since 2020. Therefore, its use in the current study was limited. Therefore, ARNi was pooled with ACEi and ARB. ^§^ SGLT2i was covered by medical insurance for heart failure indication since 2020. Therefore, its use in the current study was limited. ^¶^ No, single, double, triple and quadruple therapies of GDMT for HFrEF indicated the use of none, anyone, any two, any three or all four drugs of beta-blocker, ACEi/ARB/ARNi, MRA and SGLT2i. ACEi = angiotensin-converting enzyme inhibitor; ARNi = angiotensin receptor-neprilysin inhibitor; ARB = angiotensin II receptor blocker; HFmrEF = heart failure with mildly reduced ejection fraction; HFrEF = heart failure with reduced ejection fraction; GDMT = guideline-directed medical therapy; MRA = mineralocorticoid receptor antagonist; NOAC = non-vitamin K antagonist oral anticoagulants; SGLT2i = sodium-glucose co-transporter 2 inhibitor.

## Data Availability

The data that support the findings of this study are available from the corresponding author upon reasonable request.

## Supplementary Materials

The following supporting information can be downloaded at: https://www.mdpi.com/article/10.3390/diagnostics13213325/s1, Figure S1: Time-to-event curves comparing AF patients with HFrEF and HFmrEF who underwent the combined RFCA+LAAC procedure. Figure S2: Time-to-event curves comparing between the procedural and non-procedural cohorts of AF-HFrEF patients. Figure S3: Time-to-event curves comparing between the procedural and non-procedural cohorts of AF-HFmrEF patients. Table S1: The inclusion and exclusion criteria for the LAACablation registry; Table S2: Baseline characteristics between HF and AF patients underwent the combined RFCA+LAAC procedure and no procedure.
