# Supplementary material for: Combined Radiofrequency Ablation and Left Atrial Appendage Closure in Atrial Fibrillation and Systolic Heart Failure
Source: Diagnostics (Basel). 2023 Oct 26;13(21):3325. doi: 10.3390/diagnostics13213325 (PMC10647617; doi:10.3390/diagnostics13213325)
Supplement: Supplementary file 1 [file diagnostics-13-03325-s001.zip › diagnostics-2624005-supplementary.pdf]

Supplementary Materials

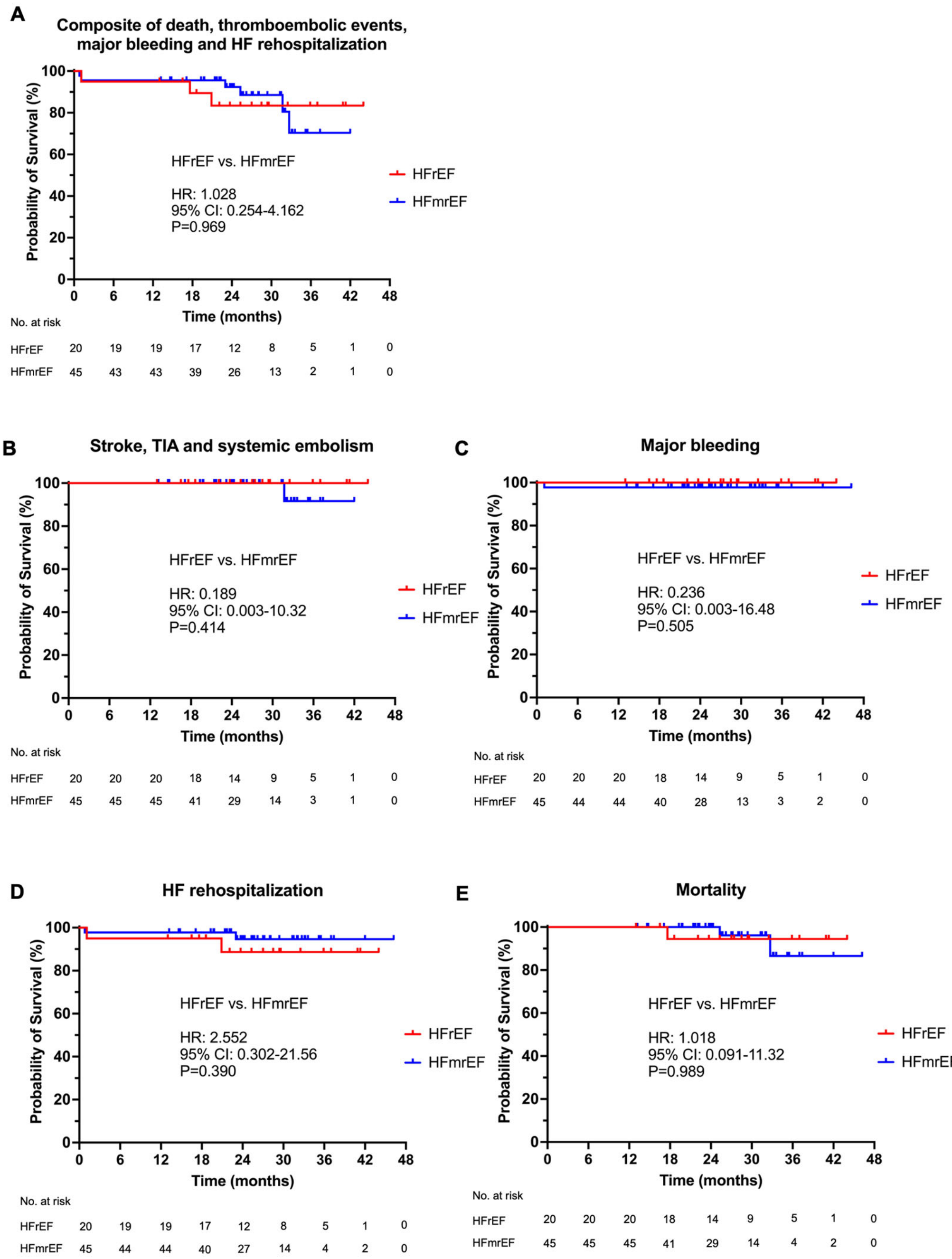

Figure S1. Time-to-event curves comparing AF patients with HFrEF and HFmrEF who underwent the combined RFCA+LAAC procedure. Time-to-event curves comparing AF patients complicating with HFrEF and HFmrEF who underwent combined RFCA and LAAC procedure in LAACablation registry. (A). Cumulative incidence of the composite of thromboembolic

events, major bleeding, heart failure rehospitalization, and death. (B). Cumulative incidence of thromboembolic events (strokes, transient ischemic attacks, and systemic embolism). (C). Cumulative incidence of major bleeding. (D). Cumulative incidence of heart failure rehospitalization. (E). Cumulative incidence of death. CI=confidence interval; HF=heart failure; HFmrEF= heart failure with mildly-reduced ejection fraction; HFrEF= heart failure with reduced ejection fraction; HR= hazard ratio; LAAC= left atrial appendage closure; RFCA= radiofrequency catheter ablation; TIA= transient ischemic attack.

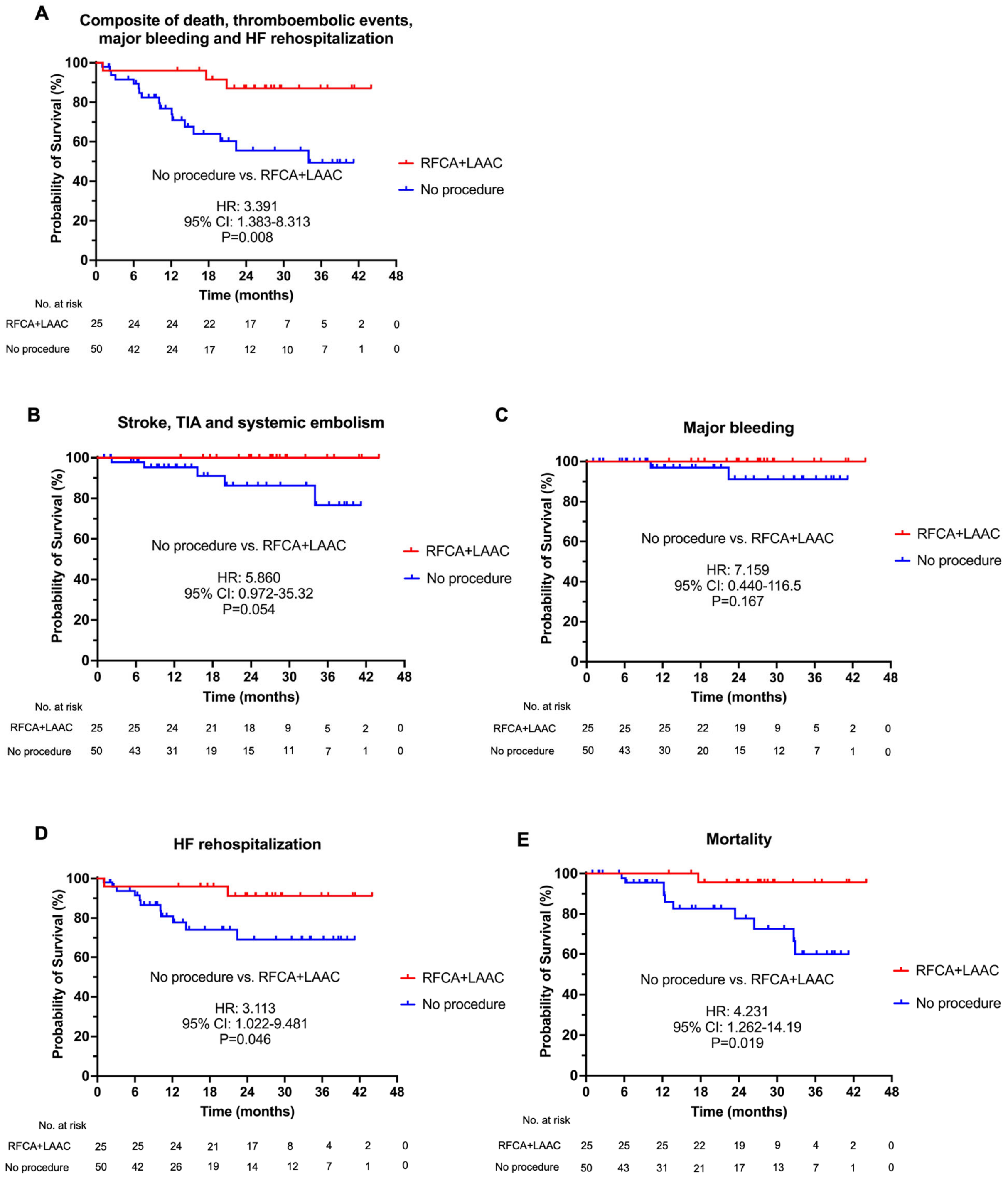

Figure S2. Time-to-event curves comparing between the procedural and non-procedural cohorts of AF-HFrEF patients. (**A**)**.** Cumulative incidence of the composite of thromboembolic events, major bleeding, heart failure rehospitalization, and death. (**B**)**.** Cumulative incidence of

thromboembolic events (strokes, TIA, and systemic embolism). (**C**)**.** Cumulative incidence of major bleeding. (**D**)**.** Cumulative incidence of heart failure rehospitalization. (**E**)**.** Cumulative incidence of death. CI=confidence interval; HF=heart failure; HFrEF= heart failure with reduced ejection fraction; HR= hazard ratio; LAAC= left atrial appendage closure; RFCA= radiofrequency catheter ablation; TIA= transient ischemic attack.

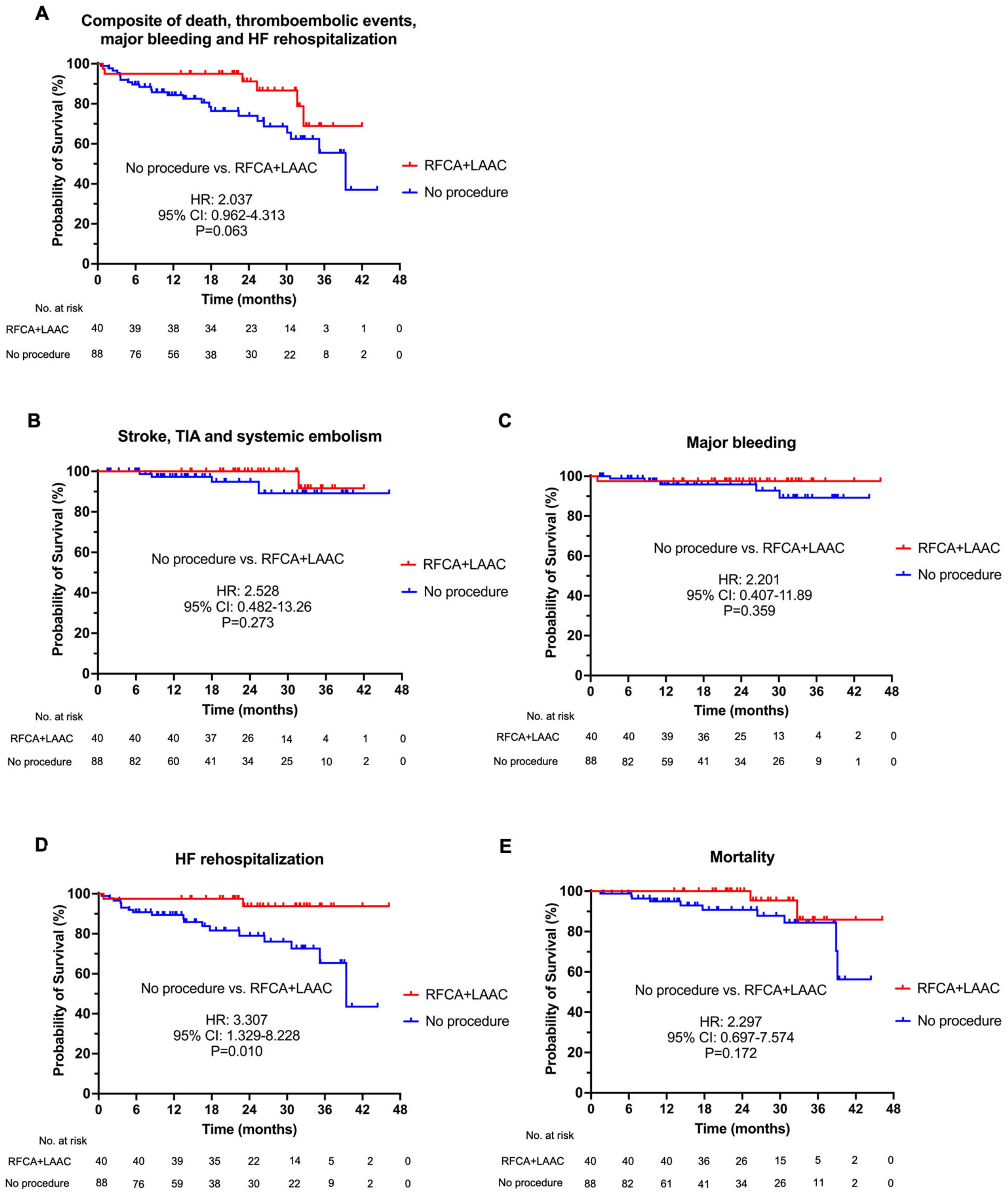

Figure S3. Time-to-event curves comparing between the procedural and non-procedural cohorts of AF-HFmrEF patients. (A). Cumulative incidence of the composite of thromboembolic events, major bleeding, heart failure rehospitalization, and death. (B). Cumulative incidence of

thromboembolic events (strokes, TIA, and systemic embolism). (C). Cumulative incidence of major bleeding. (D). Cumulative incidence of heart failure rehospitalization. (E). Cumulative incidence of death. CI=confidence interval; HF=heart failure; HFrEF= heart failure with reduced ejection fraction; HR= hazard ratio; LAAC= left atrial appendage closure; RFCA= radiofrequency catheter ablation; TIA= transient ischemic attack.

Table S1. The inclusion and exclusion criteria for the LAACablation registry.

**Inclusion criteria**
1) History of paroxysmal/persistent/longstanding persistent atrial fibrillation
2) Refractory to at least one antiarrhythmic drug, or unwilling to receive long-term antiarrhythmic medications
3) With contraindication of long-term anticoagulation, or unwilling to receive long-term anticoagulation
4) CHA_2DS_2-VASc score ≥ 2 and/or HAS-BLED score ≥ 3
5) With informed consent to participate in the study
6) Age between 18 and 90 years
**Exclusion Criteria**
1) Myocardial infarction within 3 months
2) Stroke or systemic embolism within 3 months
3) With a plan to receive heart transplantation
4) Life expectancy less than 1 year
5) Severe bleeding diseases that cannot be treated with short-term anticoagulants
6) With left atrial or left atrial appendage thrombus
7) With uncontrolled malignant tumors
8) Obvious liver and kidney dysfunction (ALT, AST more than 2 times of the upper limit of normal, or CCr <50%)
9) Women who are pregnant or breastfeeding.
10) Other conditions not suitable to receive the combined procedure evaluated by the operators.

ALT, alanine transaminase; AST, aspartate aminotransferase; CCr, creatinine clearance normalized 1.73 m^2 body surface area

Table S2. Baseline characteristics between HF and AF patients underwent the combined RFCA+LAAC procedure and no procedure.

Patient characteristics	Combined procedure (n=65)	No procedure (n=138)	P value
AF and HFrEF	20 (30.8)	54 (39.1)	0.248
AF and HFmrEF	45 (69.2)	84 (60.9)	0.248
Age	67.3 ± 7.5	69.2 ± 9.9	0.172
Age < 65 y	17 (26.2)	29 (21.0)	0.319
65 y≤ age < 75 y	36 (55.4)	77 (55.8)	0.956
Age ≥ 75y	12 (18.5)	32 (23.2)	0.446
Female	18 (27.7)	55 (39.9)	0.092
BMI (kg/m^2)	25.7 ± 3.4	24.7± 3.5	0.056
AF types			
Paroxysmal	10 (15.4)	36 (26.1)	0.089
Persistent/ long-standing persistent	55 (84.6)	102 (73.9)	0.089
CHA_2DS_2-VASc score	3.9 ± 1.2	4.2 ± 1.5	0.159
HAS-BLED score	2.7 ± 1.0	2.8 ± 0.9	0.477
NYHA class			
NYHA class II	24 (36.9)	41 (29.7)	0.304
NYHA class III	37 (56.9)	80 (58.0)	0.888
Ambulatory NYHA class IV	4 (6.2)	17 (12.3)	0.222
Hypertension	51 (78.5)	101 (73.2)	0.419
Diabetes mellites	15 (23.1)	37 (26.8)	0.199
Myocardial infarction	2 (3.1)	13 (9.4)	0.151
Cardiac revascularization†	6 (9.2)	19 (13.8)	0.359

Hypertrophic cardiomyopathy	4 (6.2)	11 (8.0)	0.778
Left ventricular noncompaction cardiomyopathy	2 (3.1)	5 (3.6)	>0.999
Obstructive sleep apnea	4 (6.2)	13 (9.4)	0.590
Chronic obstructive pulmonary disease	3 (4.6)	11 (8.0)	0.555
History of strokes/TIAs/SE	13 (20.0)	33 (23.9)	0.534
History of LAA thrombus	6 (9.2)	8 (5.8)	0.367
History of major bleeding	11 (16.9)	19 (13.8)	0.555
History of malignant tumor	4 (6.2)	18 (13.0)	0.225
History of chemotherapy	3 (4.6)	14 (10.1)	0.278
Pacemaker implantation	3 (4.6)	4 (2.9)	0.682
ICD/ CRT-D implantation	2 (3.1)	3 (2.2)	0.656
Current smoker	23 (35.4)	55 (39.9)	0.541
Echocardiographic parameters			
LVEF (%)	42.6 ± 5.3	41.3 ± 4.6	0.075
Left atrial diameter (mm)	47.1 ± 5.0	48.2 ± 4.1	0.099
LVEDD (mm)	57.0 ± 6.6	57.2 ± 5.7	0.825
LVESD (mm)	44.1 ± 6.6	45.4 ± 5.8	0.156
Estimated PASP (mmHg)	34.3 ± 9.3	35.8 ± 7.4	0.218
Pericardial effusion, mm‡	2.6 ± 1.2	2.8 ± 0.9	0.188
NT-proBNP (pg/ml)	1492 [809, 3259]	1633 [833, 3713]	0.481
Troponin I (ng/ml)	0.019 [0.009, 0.033]	0.021 [0.010, 0.040]	0.718
Hemoglobin, g/L	136 ± 14	133 ± 11	0.099
Creatine, mmol/L	76 ± 19	80 ± 15	0.106

Values are given as mean ± SD, median [25th percentile, 75th percentile], or n (%) as appropriate.

AF= atrial fibrillation; BMI= body mass index; CRT-D= cardiac resynchronization therapy with defibrillation;

ICD= implantable cardioverter defibrillator; LAA= left atrial appendage; LAAC= left atrial appendage closure; LVEDD= left ventricular end-diastolic diameter; LVEF= left ventricular ejection fraction; LVESD= left ventricular end-systolic diameter; NT-proBNP= N-terminal-proB-type natriuretic peptide; NYHA= New York Heart Association; PASP= pulmonary artery systolic pressure; RFCA= radiofrequency catheter ablation; SE= systemic embolism; TIA= transient ischemic attack;
